# Rehabilitation of Posterior Maxilla with Obturator Supported by Zygomatic Implants

**DOI:** 10.1155/2018/3437417

**Published:** 2018-04-23

**Authors:** Sankalp Mittal, Manoj Agarwal, Debopriya Chatterjee

**Affiliations:** ^1^Department of Oral and Maxillofacial Surgery, Government Dental College (RUHS-CODS), Jaipur, India; ^2^Department of Conservative, Government Dental College (RUHS-CODS), Jaipur, India; ^3^Department of Periodontics, Government Dental College (RUHS-CODS), Jaipur, India

## Abstract

Prosthetic rehabilitation of atrophic maxilla and large maxillary defects can be done successfully by zygomatic implant-supported prosthesis. Zygomatic implants are an avant-garde to complex and invasive-free vascularised osteocutaneous flaps, distraction osteogenesis, and the solution to flap failures. A treated case of tuberculous osteomyelitis, with a class II (Aramany's classification) maxillary defect, reported to oral maxillofacial department, Government Dental College (RUHS-CODS). The defect in this group was unilateral, retaining the anterior teeth. The patient was previously rehabilitated with a removable maxillary obturator. Inadequate retention affected essential functions like speaking, mastication, swallowing, esthetics, and so on due to lack of sufficient supporting tissues. A fixed prosthetic rehabilitation of posterior maxillary defect was done with obturator supported with two single-piece zygomatic implants. At 1-year follow-up, the patient was comfortable with the prosthesis, and no further complaints were recorded.

## 1. Introduction

Extensive maxillary defect and atrophic maxilla pose a challenge for prosthetic rehabilitation. Procedures for rehabilitation of large maxillary defect involve Le Fort I maxillary downfracture, onlay bone grafts, maxillary sinus graft procedures, and free osteocutaneous flaps. These procedures mostly require a second surgery at the donor site. Zygomatic implants provide an effective means to avoid the second surgical site and still rehabilitate the patient with a fixed prosthesis.

## 2. Case Presentation

**Figure 1:**
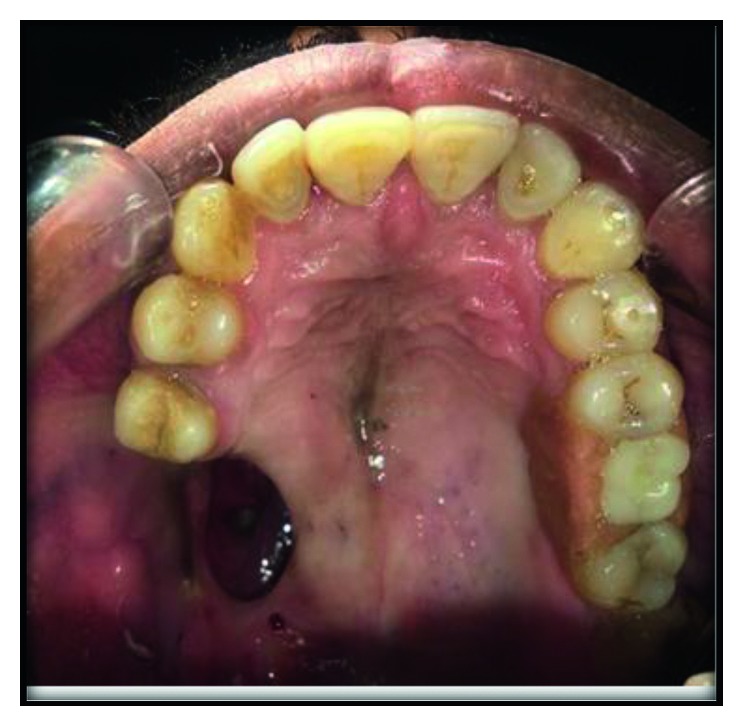
Preoperative intraoral view showing class II defect according to Armany's classification of maxillectomy defect.

A 42-year-male was referred to the oral maxillofacial department of Government Dental College and Hospital (RUHS-CODS), Jaipur with complaint of difficulty in speaking, swallowing, chewing, and visible appearance. There was a history of maxillectomy (class II defect according to Armany's classification of maxillectomy defect) of the right side posterior to the second premolar two years back due to tuberculosis osteomylitis (Figure 1). The defect categorized in this group is unilateral, retaining the anterior teeth [1].

Six months after maxillectomy, the patient was rehabilitated with hollow bulb obturator. The maxillary obturator was not stable. The use of conventional dental implants was restricted as the defect size was large; adequate teeth for retention were not present, and underlying bone support was inadequate. The zygomatic implant was planned to address these issues and to rehabilitate the posterior maxillary defect.

A detailed case history was elicited with thorough extraoral and intraoral examination. Evaluation was done with respect to bone volume and density of the remaining zygomatic bone, intermaxillary relationship, occlusal relationship, and the condition of opposing dentition.

**Figure 2:**
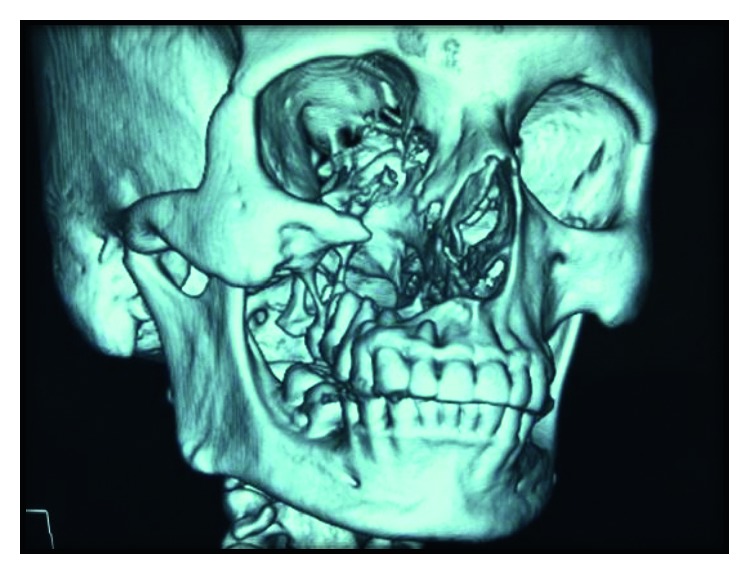
Preoperative 3-DCT view showing the missing body of the maxilla on the right side along with the lateral border of pyriform aperture and medial infraorbital margin.

**Figure 3:**
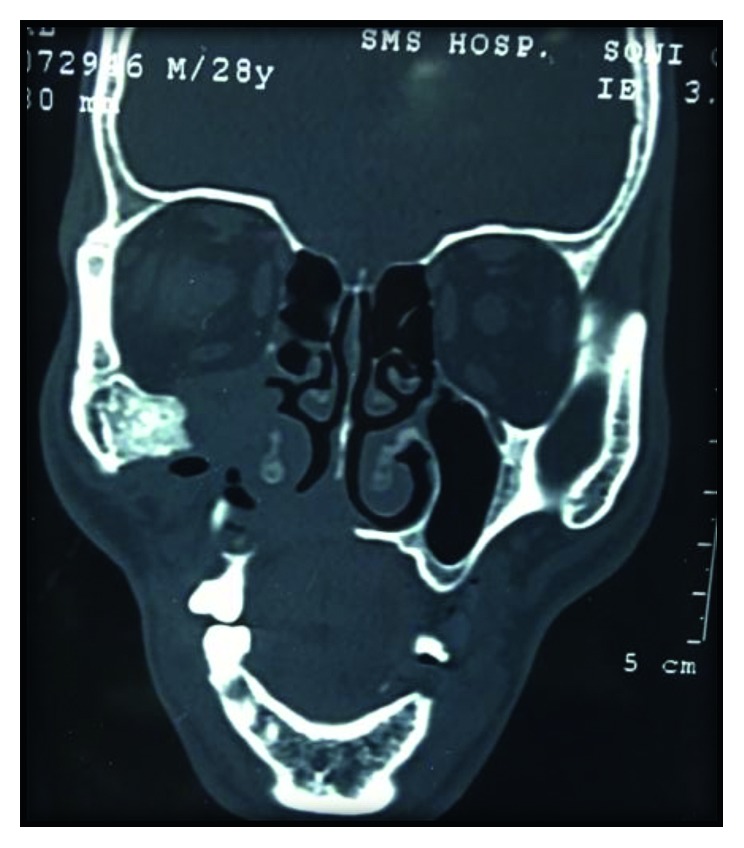
Axial CT view showing maxillectomy defect.

The 3-D computed tomography (CT) examination showed that the body of the maxilla on right side along with lateral border of pyriform aperture and medial infraorbital margin was missing (Figures 2 and 3). On clinical examination, 1.5 × 2.0 cm defect was present posterior to second premolar along with oroantral communication.

**Figure 4:**
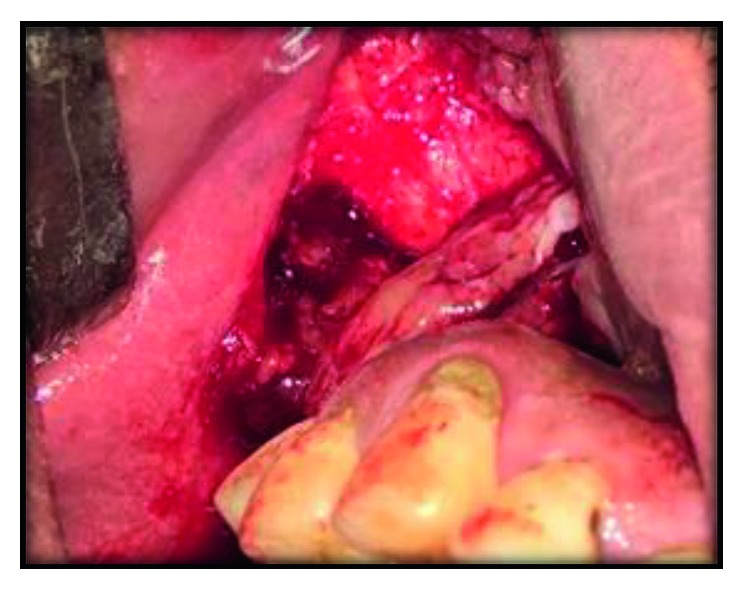
Mucoperiosteal flap elevated up to the zygomatic bone.

It was decided to use single-piece zygomatic implants (IHDE Dental, Sewden). The surgery was performed under general anesthesia. A modification of the traditional lefort 1 incision^9^ was made, where in the incision was given in the right lateral buccal mucosa in the zygomatic buttress region. Mucoperiosteal flap was elevated up to the zygomatic bone (Figure 4).

**Figure 5:**
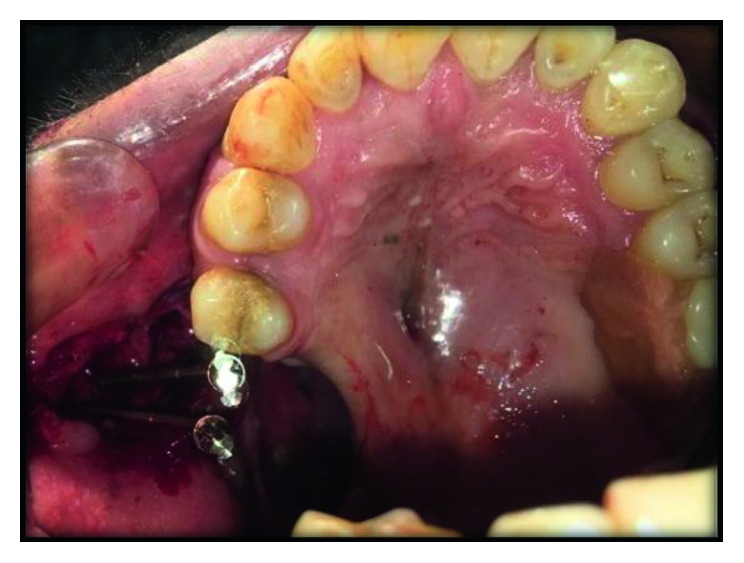
Intraoral view of two standard zygomatic single-piece implants placed in right zygomatic bone.

Two standard zygomatic single-piece implants were placed in the right zygomatic bone (Figure 5). The lengths were 47.5 and 45 mm. Horizontal mattress sutures were placed to close the mucoperiosteal flap. Silk sutures were used.

An antibiotic (amoxicillin and potassium clavulanate 625 mg TDS) was prescribed for 10 days postoperatively. Analgesic was prescribed. Sutures were removed after 15 days. A soft diet was advised for the first 2 weeks. Both the implants demonstrated good primary stability.

**Figure 6:**
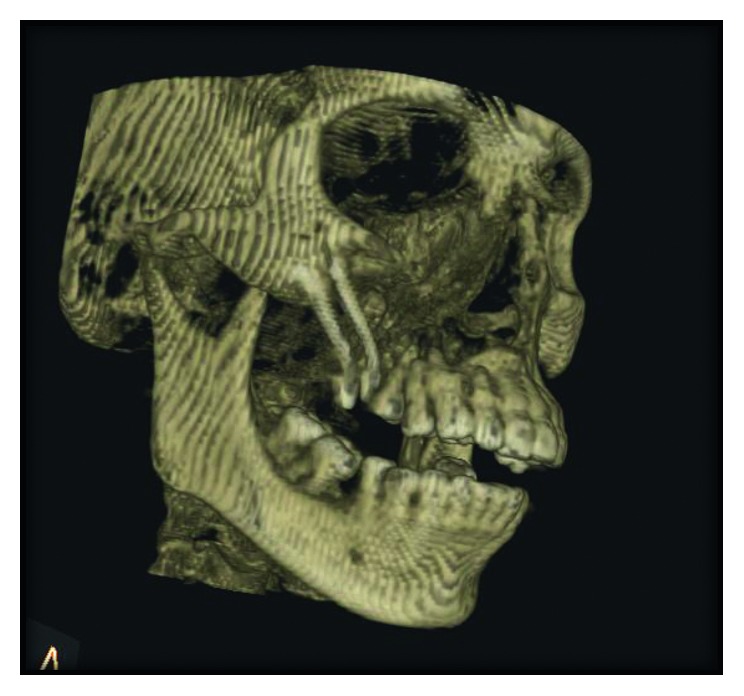
Postoperative CT shows apical two-thirds of zygomatic implant has engaged the medullary and cortical regions of the zygomatic bone.

Postoperative CT showed that apical two-thirds of the zygomatic implant has engaged the medullary and cortical region of the zygomatic bone (Figure 6).

Zygomatic implant-supported prosthetic rehabilitation was initiated 3 weeks after placement of the implants. A wax pattern of the obturators was fabricated. Retention was mainly derived from the zygomatic implants, with support from the residual horizontal palatal bone.

**Figure 7:**
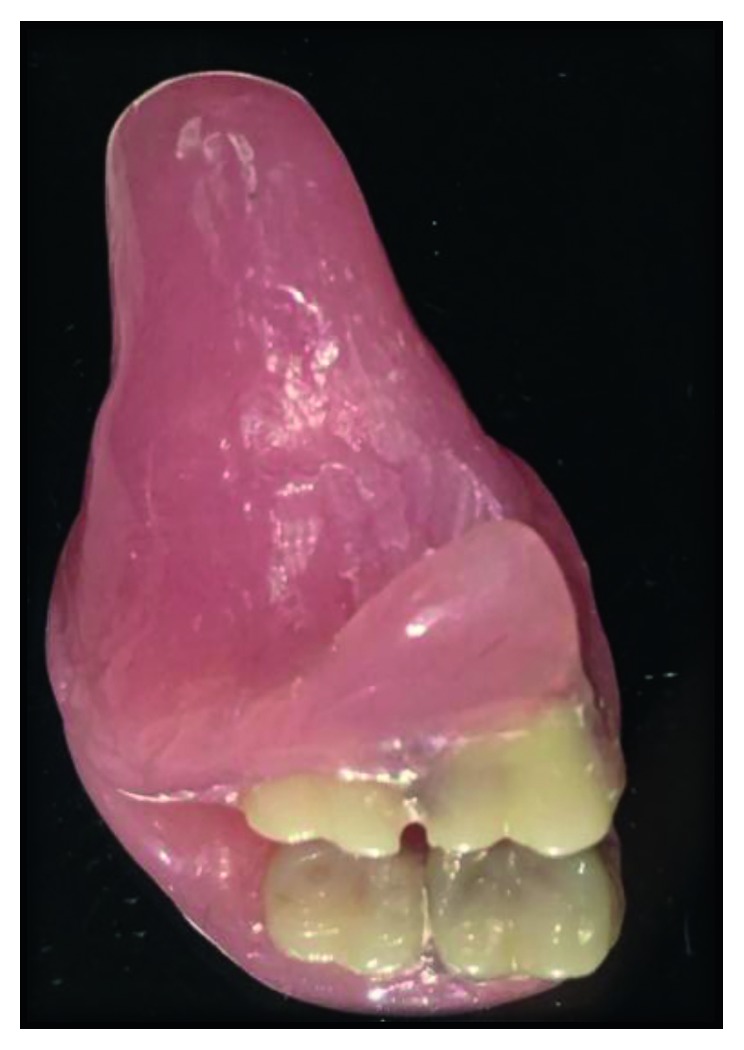
Acrylised obturator.

The implant head copings were transferred to the obturator, and fit was checked on the master cast. The model was then mounted in an articulator to check for interarch occlusal relationship after arrangement of teeth. Trial insertion was done. The whole assembly was acrylised (Figure 7) and returned to the articulator for final occlusal equilibration.

**Figure 8:**
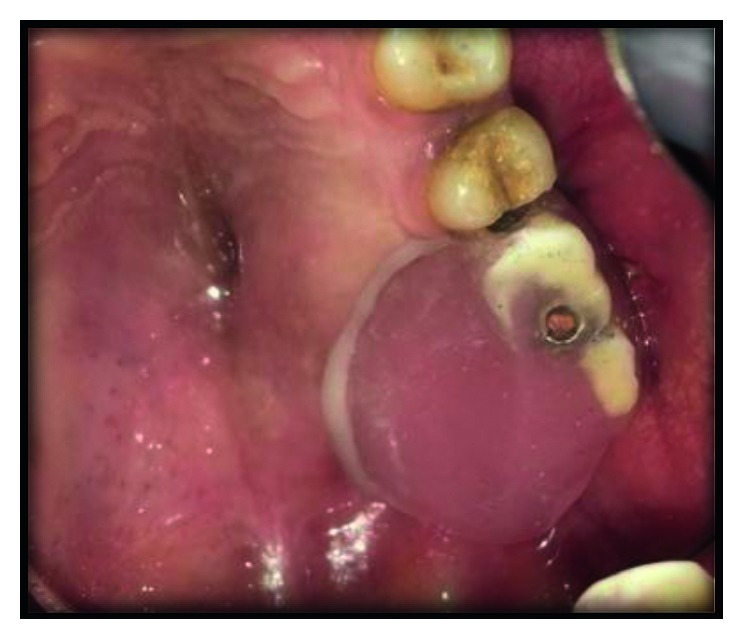
obturator supported by two zygomatic implants.

Following insertion of the prosthesis (Figure 8), the obturator prosthesis demonstrated optimal retention and stability. Postinsertion instructions were given with focus on insertion, removal, and hygiene of the prosthesis. At 1-year follow-up, the patient confirmed his satisfaction and no significant complaints have been recorded. The patient's phonation, chewing, deglutition, and aesthetic improved.

## 3. Discussion

Maxillary posterior defects that occur due to tumor resection, trauma, or any pathologic lesion pose a challenge to reconstruct and rehabilitate. The aim of rehabilitation is to provide a cosmetically acceptable appearance and to restore functions [2].

Defects in posterior maxilla have been reconstructed with invasive and lengthy procedures such as onlay grafts, free or microvascular bone grafts, and transport distraction osteogenesis with or without a Le Fort I osteotomy with a success rates of 60–90% [3, 4].

Many factors affect the retention of the maxillary obturator example, size of the defect, the number of remaining teeth, the amount of the remaining bony structure, and the patient ability to adapt to the prosthesis [5].

Most of the cases with maxillectomy have limited bone support and large oral and facial soft tissue defects. Implant-supported or conventional obturator prosthesis is extremely complicated in these cases [6].

The introduction of dental implants in obturator brings wonderful improvement in the performance of obturator by exhibiting better mechanical qualities [7, 8].

Placement of the conventional endosteal implant would compromise long-term osseintegration. Limited support from the remaining anatomic structures results in placement of the endoosseous implant at an increased angle; hence, prosthetic rehabilitation becomes difficult [9].

Hence prosthetic rehabilitation of posterior maxillary defect with obturator supported with two single-piece zygomatic implants was done.

The zygomatic implant, first introduced by Brånemark in 1988, is especially for patients with atrophy of the maxilla or who suffer from a complication after bone grafting procedures [10].

Main indications of the zygomatic implant are significant sinus pneumatization and alveolar ridge resorption. Contraindications to the use of zygomatic implants include acute sinus infection, any kind of pathology of maxillary and zygomatic bone, and underlying uncontrolled or malignant systemic disease [11]. The zygomatic bone is pyramid in shape and contains dense cortical and trabecular bones. According to a cadaver study, the mean length of available bone in this region is about 14 mm. The bone length is sufficient for placement of the zygomatic implant in patients with severely resorbed posterior maxillary bone [12].

The original Branemark zygomatic implant was a self-tapping titanium implant with a treated surface, available in lengths of 30–52.5 mm. The diameter at the threaded apical part was 4 mm and 4.5 mm at the crestal part. The implant head was provided with an inner thread for connection of standard abutments.

At present, there are three different companies that offer zygomatic implants with an oxidized rough surface, a smooth midimplant body, a wider neck at the alveolar crest, and a 55° angulation of the implant head [12].

The zygomatic bone has thick cortical layer that offers a solid and extended anchorage that can bear the vertical masticatory forces. The tricortical anchorage increases the success and survival of the zygomatic implant [13].

In the present case, two zygomatic implants provided sufficient retention and support for the obturator.

The greatest advantage of the zygomatic implant is the elimination of donor site morbidity and infection in graft material.

There is literature available on zygomatic implant, but till date, no randomized controlled trials comparing the clinical outcomes of zygomatic implants with alternative means for rehabilitating patients with atrophic edentulous maxilla are available [14].

Although limited clinical data are available on the long-term clinical performance of zygomatic implants, the present case report provides evidence that the zygomatic implant is an adaptable option to rehabilitate wide maxillary defects.
